# Long non-coding RNA TTN-AS1/microRNA-199a-3p/runt-related transcription factor 1 gene axis regulates the progression of oral squamous cell carcinoma

**DOI:** 10.1080/21655979.2021.1982324

**Published:** 2021-10-04

**Authors:** Zhongzhi Jin, Shengjun Jiang

**Affiliations:** Department of Stomatology, Renmin Hospital of Wuhan University, Wuhan, Hubei, China

**Keywords:** TTN-AS1, miR-199a-3p, RUNX1, oral squamous cell carcinoma

## Abstract

Oral squamous cell carcinoma (OSCC) has a high degree of malignancy, which affects the quality of life and prognosis of patients with OSCC. Our study aimed to reveal the function of long non-coding RNA TTN-AS1/microRNA-199a-3p (miR-199a-3p)/runt-related transcription factor 1 (RUNX1) axis in OSCC progression, thereby providing a novel OSCC effective strategy. Real-time quantitative polymerase chain reaction and western blotting were performed to detect the expression of TTN-AS1, miR-199a-3p, and RUNX1 in OSCC. Several cell functional experiments, including Cell Counting Kit-8, flow cytometry, and cell adhesion assays, were used to assess cell proliferation, apoptosis, adhesion, and migration. A luciferase assay was performed to confirm the interaction between TTN-AS1, miR-199a-3p, and RUNX1. Our results revealed that TTN-AS1 and RUNX1 were upregulated in OSCC tissues and cells, whereas miR-199a-3p expression was downregulated. Knockdown of TTN-AS1 or RUNX1 suppressed cell proliferation, adhesion, and migration but induced apoptosis. Additionally, miR-199a-3p inhibitor partly relieved the effects of silencing TTN-AS1 and RUNX1 in OSCC cells due to their targeting relationship. In conclusion, TTN-AS1 and RUNX1 could promote OSCC progression and miR-199a-3p partly relieved the effects of TTN-AS1 and RUNX1.

## Introduction

Oral squamous cell carcinoma (OSCC) occurring in the head and neck has a high degree of malignancy, leading to a poor prognosis [1]. There are >300,000 new cases of OSCC every year worldwide, and over 140,000 OSCC patients die annually [2,3]. The causes of OSCC mainly include smoking, drinking, and ultraviolet radiation [4]; therefore, people with a long history of smoking tobacco and drinking alcohol are susceptible to OSCC [5]. Patients with OSCC often present with fissuring ulcers, lumps, red or white lesions, and cervical lymph node enlargements [6]. For definitive diagnosis of OSCC, surgical biopsy and histopathological examination remain the gold standard [7]. Although various treatment methods, including surgical resection, radiotherapy, and chemotherapy, are widely applied in clinics, the life quality and prognosis of OSCC (50% 5-year survival rate) patients are still unsatisfactory [1]. Therefore, the molecular mechanism of OSCC progression is crucial for exploring effective OSCC strategies.

Long non-coding RNAs (lncRNAs), a group of non-coding RNAs >200 nt in length, are considered as by-products of RNA polymerase II during transcription [8,9]. An increasing number of studies have reported that lncRNAs can participate in the progression of cancer [10–12]. In OSCC, lncRNA UCA1 has been shown to enhance the proliferation and cisplatin resistance of OSCC [13]. The lncRNA CASC9 has also been reported to be a tumor promoter in OSCC [1]. The lncRNA Titin antisense RNA 1 (TTN-AS1) is transcribed from the opposite strand of the Titin (TTN) gene, and its overexpression correlates with poor prognosis in multiple cancers, including breast cancer, lung cancer, and reproductive system cancers [14]. In OSCC, TTN-AS1 was found to be upregulated and exerted a positive function in OSCC by targeting the miR-411-3p/NFAT5 axis [15]. Owing to the complex regulatory mechanism of TTN-AS1 in breast cancer by regulating the miR-524-5p/RRM2 axis [16] or miR-139-5p/ZEBI axis [17], the effect of TTN-AS1 on OSCC progression might involve other miRNAs or mRNAs, which needs to be explored further.

miRNAs, a type of single-chain small molecule RNAs, can be sponged by lncRNAs to target their target genes, thereby regulating the progression of cancers [18–20]. miR-199a-3p, a member of the miRNA family, has been shown to play different roles in different cancer types. For instance, miR-199a-3p promotes gastric cancer cell invasion and migration [21], but it has an anti-tumor effect on ovarian cancer [22]. In OSCC, miR-199a-5p was found to suppress OSCC progression by targeting SOX4 [23]. However, the function of miR-199a-3p in OSCC has not been explored.

Runt-related transcription factor 1 (RUNX1) can encode transcription factors that bind to DNA in partnership with core-binding factor β (CBFβ), thereby enhancing DNA-binding activity and stability [24–26]. In head and neck squamous cell carcinoma (HNSCC), RUNX1 with high expression was associated with tumorigenicity of HNSCC [27]. In 2012, RUNX1 with high expression was observed in oral cancer cells and was found to be responsible for oral tumor formation [28]. However, the mechanism of OSCC progression has not been explored so far.

In this study, we suspected that the TTN-AS1/miR-199a-3p/RUNX1 axis plays an important role in OSCC. Hence, we aimed to explore the function of the TTN-AS1/miR-199a-3p/RUNX1 axis in OSCC using bioinformatics analysis and cell functional experiments. Our findings provide novel insights into OSCC therapy.

## Material and methods

### Clinical sample collection and cell culture

OSCC tissues and adjacent normal tissues were collected from 36 OSCC patients (age range: 46–72 years, 23 males and 13 females) from our hospital between April 2018 and April 2020. All patients were asked to complete written informed consent forms, and their clinical characteristics are shown in Supplementary Table 1. Our study was approved by the Ethics Committee of our hospital.

The human oral epithelial cell line (HOEC) and OSCC cell lines (SCC-4 and CAL-27) were purchased from BeNa Culture Collection (China), and another OSCC cell line, HSC-3, was a gift from the State Key Laboratory of Oral Diseases, Sichuan University (China). SCC-4 cells were cultured in 90% EMEM medium and 10% FBS, whereas the other cells were cultured in 90% DMEM and 10% FBS. All cells were kept in an incubator at 37°C and 5% CO_2_.

### Real-time quantitative polymerase chain reaction (RT-qPCR)

The HiPure Total RNA Mini Kit (Magen, China) was used to isolate RNA from tissues and cells to detect lncRNA and mRNA. Total RNA was reverse transcribed and RT-qPCR was performed using the BeyoFast SYBR Green One-Step RT-qPCR Kit (Beyotime, China). Total RNA was isolated from tissues and cells using an miRNA extraction kit (HaiGene, China). Reverse transcription and RT-qPCR were performed using the BeyoFast Probe One-Step RT-qPCR Kit (Beyotime, China). The 2^−ΔΔCt^ method [29] was used to calculate the relative expression of lncRNA, mRNA, and miRNA using GAPDH as the internal reference of lncRNA and mRNA, and U6 as the internal reference of miRNA. The primer sequences are listed in Supplementary Table 2.

### Cell transfection

Two siRNAs of TTN-AS1 (si-lnc1 and si-lnc2), miR-119a-3p mimic/inhibitor, siRNA of RUNX1 (si-RUNX1), and their negative control (NC), including si-NC, mimic-NC, and inhibitor-NC, were purchased from Tuoran Co., Ltd. (China). First, 2 × 10^5^ SCC-4 and HSC-3 were seeded into 24-well plates for incubation overnight. Then, 50 nM si-lnc1, si-lnc2, miR-119a-3p mimic/inhibitor, si-RUNX1, and their NC were transfected into cells using Lipofectamine 2000 (Invitrogen, USA) for 48 h. The detection of transfection efficiency and subsequent experiments were performed 48 h after transfection.

### Cell counting kit-8 (CCK8) assay

OSCC cells (5,000 cells in 100 μL medium per well) after 48 h of transfection were seeded into 96-well plates. After incubation for 0, 24, 48, and 72 h, 10 μL of CCK8 solution (Cat#: K1018; APExBIO, China) was added to the wells and incubated for another 4 h. The optical density (OD) values at 450 nm were measured using a multimode plate reader (Thermo, USA) to assess cell proliferation according to a previous study [30].

### Flow cytometry

Flow cytometry (BD Biosciences, USA) was performed to assess the cell apoptosis ability of transfected OSCC cells using the Annexin V-FITC/PI Apoptosis kit (Cat#: 556,547; BD, USA) according to a previous study [31]. After 48 h transfection, 1 × 10^6^ SCC-4 and HSC-3 cells were collected in 100 μL of binding buffer. Then, the cells were incubated with 10 μL Annexin V-FITC and 10 μL propidium iodide (PI) at 22–25°C in the dark. The cell apoptosis rate was detected by flow cytometry and analyzed by FlowJo 7.6.1 (Treestar, USA).

### Cell adhesion assay

The cell adhesion assay was performed according to the previous study [32]. The transfected OSCC cells were seeded in 96-well plates coated with 30 μL/well collagen I solution (Sigma-Aldrich, USA). Then, the transfected cells in 96-well plates were incubated with a serum-free medium for 1 h. After washing the non-adherent cells with a serum-free medium, the adherent cells were detected using the MTT cell proliferation assay kit (ATCC, USA) according to the manufacturer’s instructions. After incubating the cells with 10 μL MTT substrate for 4 h, OD values were measured at 450 nm using a multimode-plate-reader.

### Wound healing assay

The transfected OSCC cells (1 × 10^6^ cells in 2 mL medium per well) were seeded in 6-well plates and cultured until they reached 100% confluence. Then, 200 μL pipette tips were used to create artificial wounds. After cell culture in serum-free medium for 0 and 24 h, the images of wound healing were photographed using a microscope (Olympus, Japan) according to a previous study [31].

### Bioinformatics analysis

GSE37991 from GEO DataSets was used to select the differentially expressed genes (DEGs) in OSCC samples using limma 3.26.8. GO enrichment for the selected DEGs was analyzed using Metascape. The correlation between key genes and TTN-AS1 in head and neck squamous cell carcinoma samples was analyzed using GEPIA. Three databases, miRDB, TargetScan, and TarBase, were used to predict the miRNAs targeting key genes, whereas starBase was used to predict the miRNAs sponged by TTN-AS1.

### Luciferase assay

Wild-type (WT) TTN-AS1 or RUNX1 containing the binding sites for miR-199a-3p, and mutant (MUT) TTN-AS1 or RUNX1 containing the altered binding sites for miR-199a-3p were cloned into pGL3 luciferase reporter vectors by Tuoran (China). Then, these vectors were co-transfected with miR-199a-3p mimic or mimic-NC into SCC-4 and HSC-3 cells using Lipofectamine 2000. After 48 h of co-transfection, the activities of firefly and Renilla were measured using the Dual-Luciferase Reporter Assay System (Promega, USA) according to a previous study [33].

### Western blotting

According to a previous study, western blotting was performed to identify protein expression in OSCC cells [30]. First, the cells were lysed to isolate total proteins using RIPA buffer (Thermo, USA). After detecting the protein concentrations using a BCAkit (Thermo, USA), 20 µg/lane protein was separated by 10% SDS-PAGE at 100 V. Then, the separated proteins were transferred onto PVDF membranes for 2 h at 22–25°C using 5% skimmed milk. Next, the membranes were incubated with primary antibodies, including RUNX1 (Abcam, USA) and GAPDH (Abcam, USA) at 4°C overnight. The next day, the membranes were incubated with horseradish peroxidase-conjugated anti-rabbit IgG antibody (Abcam, USA) for 3 h. Finally, the membranes were incubated with SuperEnhanced Chemiluminescence Detection Reagent (Applygen, China) for 10 min, covered with plastic wrap, and exposed to X-ray film.

## Statistical analysis

GraphPad Prism 8.0 (GraphPad Prism, USA) was utilized to analyze the data from three repeated biological experiments that were shown as mean ± SD. The statistical difference of two groups and multiple groups were analyzed by student’s t-test and ANOVA, respectively. P-value <0.05 was considered statistically significant.

## Results

In this study, we aimed to explore the regulatory mechanisms of TTN-AS1, miR-199a-3p, and RUNX1 on OSCC progression in vitro. RT-qPCR revealed that TTN-AS1 and RUNX1 were upregulated in OSCC, and miR-199a-3p was downregulated in OSCC. Furthermore, A series of functional cell experiments proved that silencing TTN-AS1 or RUNX1 impaired cell proliferation and cell migration but enhanced cell apoptosis. Meanwhile, miR-199a-3p inhibitor partially recovered the negative effects of silencing TTN-AS1 or RUNX1 on OSCC cells. In addition, we confirmed the interaction between TTN-AS1, miR-199a-3p, and RUNX1 in OSCC cells. Overall, our study found that TTN-AS1 contributed to the malignancy of OSCC cells by sponging the miR-199a-3p/RUNX1 axis.

### Silencing TTN-AS1 inhibited the malignancy of OSCC cell

**Figure 1.**
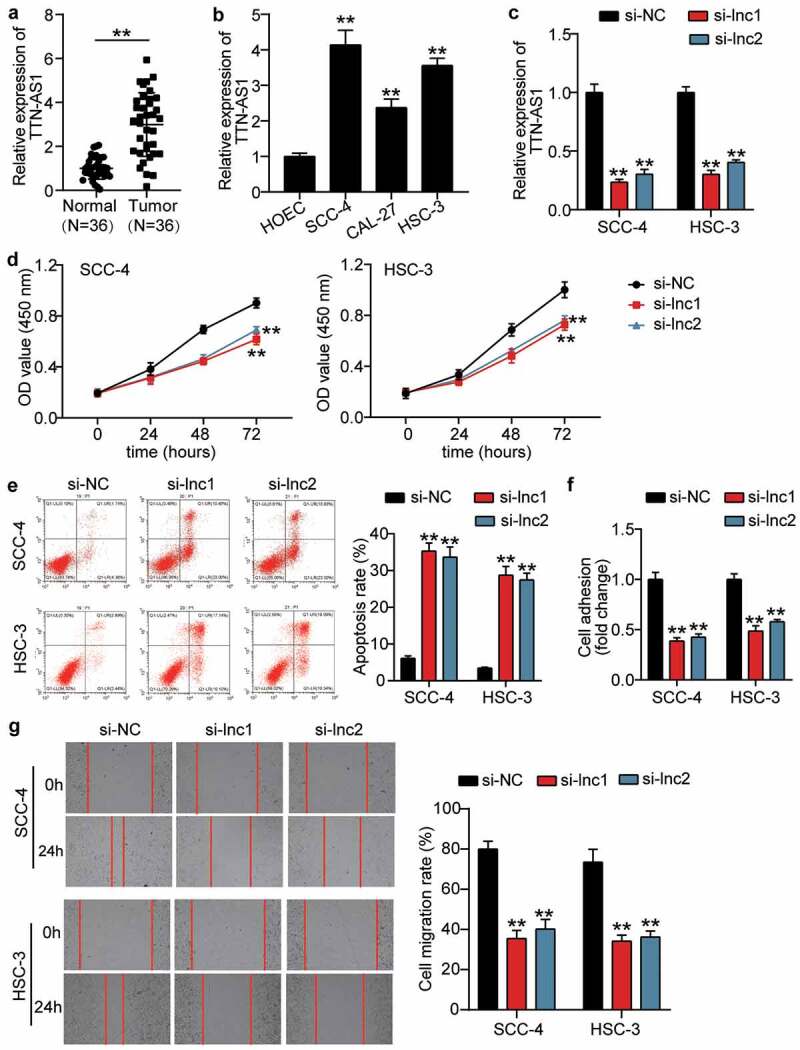
The effect of silenced TTN-AS1 on OSCC cells (a) The expression of TTN-AS1 in normal tissues and tumor tissues. Tumor, OSCC tissues. Normal, adjacent normal tissues. **P < 0.001. (b) The expression of TTN-AS1 in human oral epithelial cells (HOEC) and OSCC cells (SCC-4, CAL-27 and HSC-3). **P < 0.001 vs. HOEC. (c) The transfection efficiency of two siRNAs targeting TTN-AS1 in SCC-4 and HSC-3 cells. (d) Cell proliferation was detected using CCK8 assay in SCC-4 and HSC-3 cells transfected with si-lnc1 and si-lnc2. (e) Cell apoptosis was detected using flow cytometry in SCC-4 and HSC-3 cells transfected with si-lnc1 and si-lnc2. (f) The cell adhesion was detected using cell adhesion assay in SCC-4 and HSC-3 cells transfected with si-lnc1 and si-lnc2. (g) The migration rate was detected using wound healing assay in SCC-4 and HSC-3 cells transfected with si-lnc1 and si-lnc2. (c-g) si-lnc1 and si-lnc2 are two siRNAs of TTN-AS1. NC, negative control. **P < 0.001 vs. si-NC

To verify the influence of TTN-AS1 in OSCC, the RT-qPCR was first used to detect the expression of TTN-AS1 in OSCC tissues and cells. The results showed that TTN-AS1 was upregulated in OSCC tissues by 3-fold compared with adjacent normal tissues (Figure 1a), and TTN-AS1 expression was associated with tumor differentiation (P = 0.0010), T-stage (P = 0.0369), and lymph node metastasis (P = 0.0027) (Supplementary Table 1). In cells, TTN-AS1 expression was upregulated in OSCC cells, including SCC-4 cells (4.14 ± 0.33), CAL-24 cells (2.38 ± 0.20), and HSC-3 cells (3.564 ± 0.17), compared with human oral epithelial cells (HOEC, 1.00 ± 0.07) (Figure 1b). Because TTN-AS1 was highly expressed in SCC-4 and HSC-3 cells, two siRNAs targeting TTN-AS1 were successfully transfected into SCC-4 and HSC-3 cells (Figure 1c). The CCK8 assay revealed that silencing TTN-AS1 inhibited cell proliferation in SCC-4 and HSC-3 cells (Figure 1d). After assessing cell apoptosis by flow cytometry, it was found that the apoptosis rate was elevated in the silent TTN-AS1 groups (Figure 1e). Cell adhesion capability was impaired when SCC-4 and HSC-3 cells were transfected with two siRNAs of TTN-AS1 (figure 1f). Similar to cell adhesion, the ability of cell migration was also inhibited by transfecting two siRNAs targeting TTN-AS1 into SCC-4 and HSC-3 cells (Figure 1g). However, silencing TTN-AS1 did not affect cell adhesion and migration in HOECs (Supplementary Figure 1).

### The identification of key gene and miRNAs as the downstream of TTN-AS1

**Figure 2.**
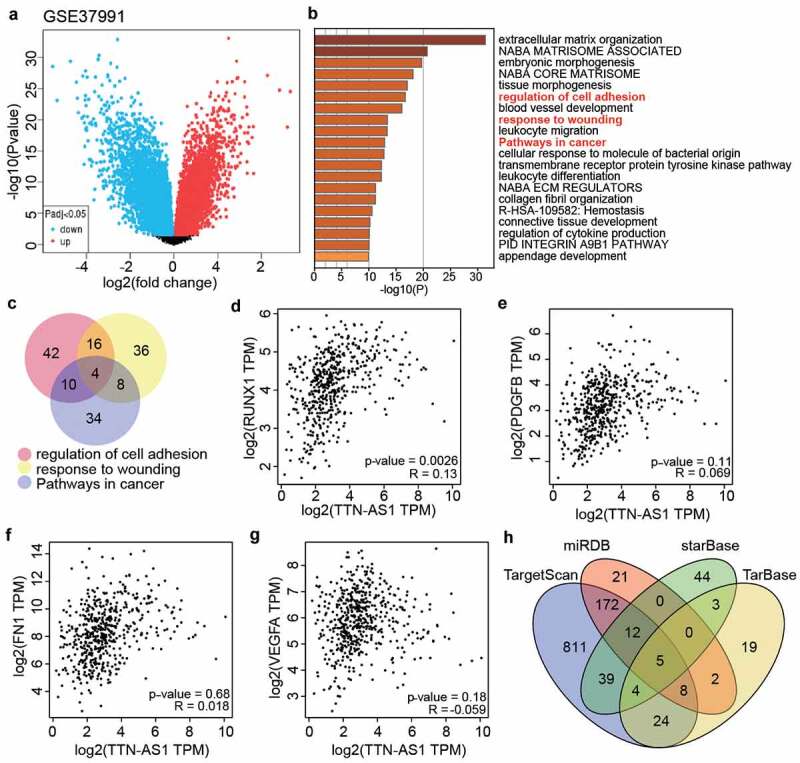
The identification of key gene and miRNAs in OSCC (a) The DEGs in OSCC samples were screened from GSE37991. (b) Metascape was used to enrich the key biological processes for the screened genes. (c) The four genes (RUNX1, PDGFB, FN1 and VEGFA) were the common genes of three biological processes. (d) The correlation between RUNX1 and TTN-AS1 in head and neck squamous cell carcinoma samples according to GEPIA data. (e) he correlation between PDGFB and TTN-AS1 in head and neck squamous cell carcinoma samples according to GEPIA data. (f) The correlation between FN1 and TTN-AS1 in head and neck squamous cell carcinoma samples according to GEPIA data. (g) The correlation between VEGFA and TTN-AS1 in head and neck squamous cell carcinoma samples according to GEPIA data. (h) Five miRNAs were the common miRNAs of miRDB, TargetScan, TarBase and starBase. miRDB, TargetScan and TarBase were three databases to predict the miRNAs targeting RUNX1. starBase was a database to predict the miRNAs sponged by TTN-AS1

GSE37991 from GEO DataSets included DEGs in the OSCC samples (Figure 2a). With adj. P < 0.05, and logFC>1, 891 upregulated DEGs were screened and uploaded to Metascape for GO enrichment. The results showed that regulation of cell adhesion, response to wounding, and pathways in cancer were enriched (Figure 2b). Using Venny 2.1.0, four genes (RUNX1. PDGFB, FN1, and VEGFA were the common genes involved in the regulation of cell adhesion, response to wounding, and pathways in cancer (Figure 2c). According to the data from GEPIA, RUNX1 expression was found to be positively related to TTN-AS1 expression in head and neck squamous cell carcinoma samples (Figure 2d-g). Therefore, we suspected that RUNX1 might be downstream of TTN-AS1. To identify the key miRNAs connecting RUNX1 and TTN-AS1, miRDB, TargetScan, and TarBase were used to predict the miRNAs targeting RUNX1, whereas starBase was used to predict the miRNAs sponged by TTN-AS1. Finally, only five miRNAs (miR-200a-3p, miR-199a-3p, miR-199b-3p, miR-27a-3p, and miR-27b-3p) overlapped with miRDB, TargetScan, TarBase, and starBase (Figure 2h).

### TTN-AS1 sponged miR-199a-3p in OSCC

**Figure 3.**
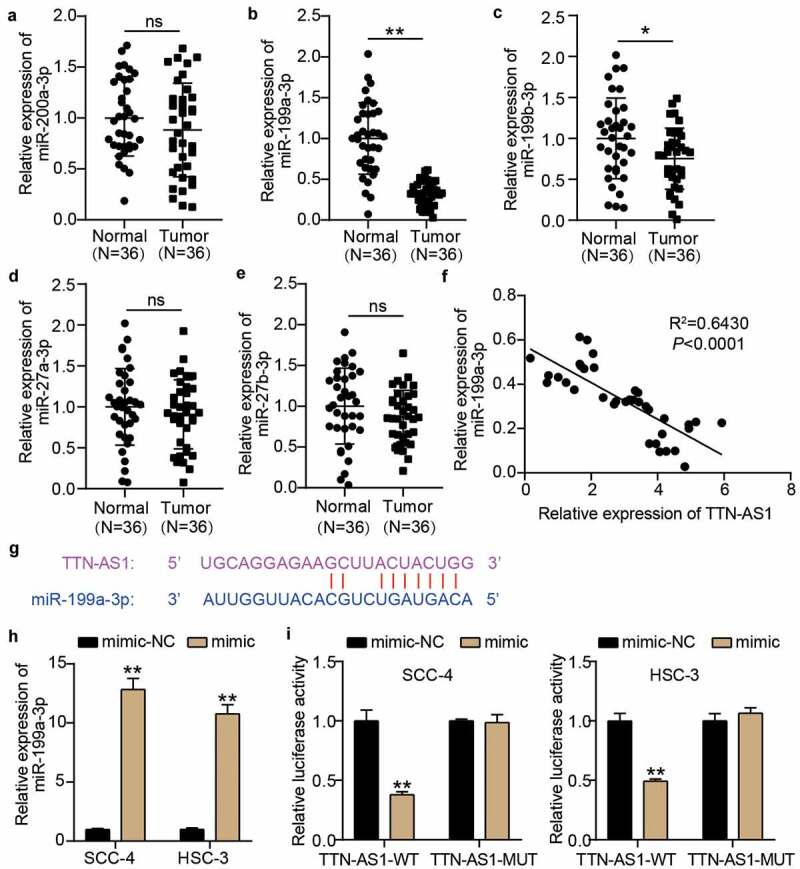
miR-199a-3p could be sponged by TTN-AS1 (a-e) The expression levels of miR-200a-3p (a), miR-199a-3p (b), miR-199b-3p (c), miR-27a-3p (d), and miR-27b-3p (e) in normal tissues and tumor tissues. Tumor, OSCC tissues. Normal, adjacent normal tissues. ns, No significant. *P < 0.05, **P < 0.001. (f) The correlation between miR-199a-3p expression and TTN-AS1 expression in OSCC tissues. (g) The binding sites between TTN-AS1 and miR-199a-3p was predicted by starBase. (h) The transfection efficiency of miR-199a-3p mimic in SCC-4 and HSC-3 cells. mimic, miR-199a-3p mimic. NC, negative control. **P < 0.001. (i) The relationship between TTN-AS1 and miR-199a-3p was identified using luciferase assay. WT, wild-type. MUT, mutant. mimic, miR-199a-3p mimic. NC, negative control. **P < 0.001

We first performed RT-qPCR to assess the expression of the five miRNAs screened by bioinformatics analysis in our collected clinical samples to identify the miRNA sponged by TTN-AS1. The results showed that miR-199a-3p was significantly downregulated in OSCC tissues compared with other miRNAs in OSCC tissues (Figure 3a-e), and miR-199a-3p expression was negatively correlated with TTN-AS1 expression in OSCC tissues (figure 3f). The binding sites between TTN-AS1 and miR-199a-3p based on the prediction of starBase are shown in Figure 3g. Then, the miR-199a-3p mimic was successfully transfected into SCC-4 and HSC-3 cells before performing the luciferase assay (Figure 3h). The luciferase assay showed that only TTN-AS1-WT and miR-199a-3p co-transfected SCC-4 and HSC-3 induced a decrease in luciferase activity, indicating that there were binding sites between TTN-AS1-WT and miR-199a-3p (Figure 3i).

### miR-199a-3p inhibitor partly relieved the effect of silenced TTN-AS1 on OSCC cells

**Figure 4.**
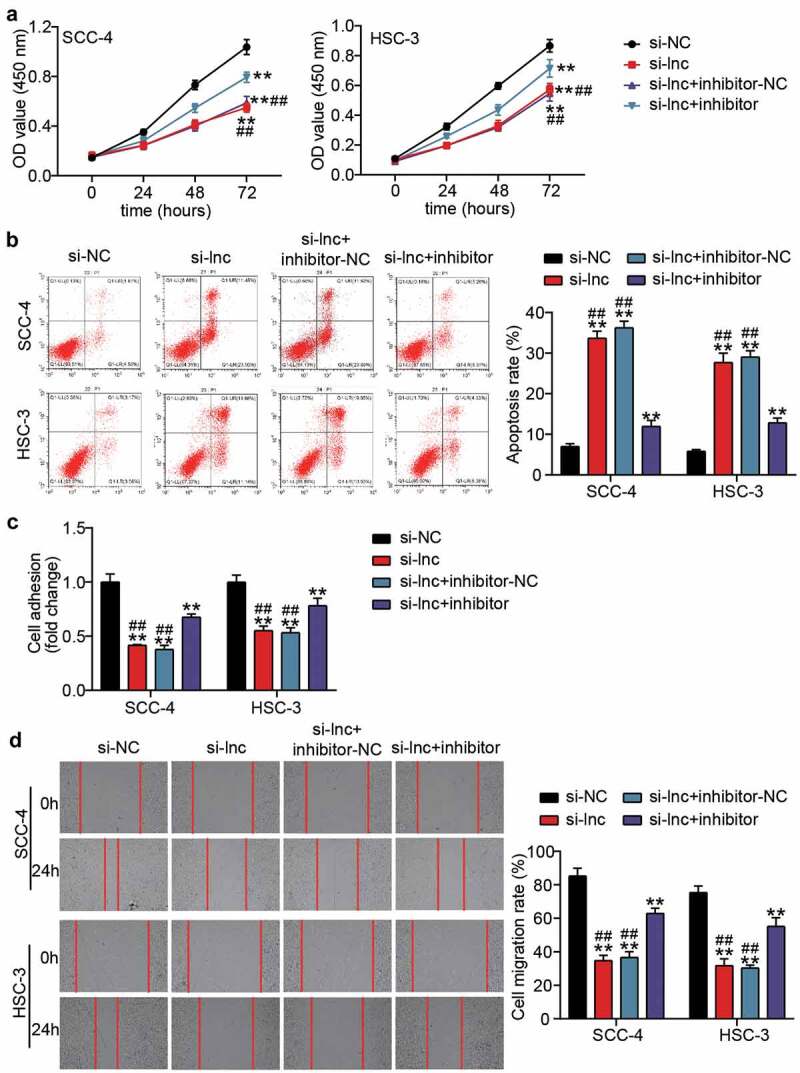
miR-199a-3p inhibitor partly relieved the effect of silenced TTN-AS1 on OSCC cells (a) miR-199a-3p inhibitor partly relieved the effect of silenced TTN-AS1 on cell proliferation. (b) miR-199a-3p inhibitor partly relieved the effect of silenced TTN-AS1 on cell apoptosis. (c) miR-199a-3p inhibitor partly relieved the effect of silenced TTN-AS1 on cell adhesion. (d) miR-199a-3p inhibitor partly relieved the effect of silenced TTN-AS1 on cell migration. si-lnc, si-TTN-AS1. inhibitor, miR-199a-3p inhibitor. NC, negative control. **P < 0.001 vs. si-NC. ##P < 0.001 vs. si-lnc+inhibitor

The miR-199a-3p inhibitor and si-lnc were co-transfected into SCC-4 and HSC-3 cells to explore the effect of miR-199a-3p on the regulation of the influence of TTN-AS1 in OSCC cells. The CCK8 assay showed that the negative effect of silencing TTN-AS1 in cell proliferation was partly relieved after co-transfection with the miR-199a-3p inhibitor (Figure 4a). After detecting cell apoptosis by flow cytometry, it was found that miR-199a-3p inhibitor decreased the high apoptosis rate caused by silencing TTN-AS1 (Figure 4b). The cell adhesion capability was inhibited by knocking down TTN-AS1, but this negative effect was partly counteracted by co-transfection with miR-199a-3p (Figure 4c). The wound-healing assay also proved that co-transfection of miR-199a-3p and si-lnc1 could elevate the migration rate, which was reduced by silencing TTN-AS1 (Figure 4d).

### RUNX1: a target of miR-199a-3p in OSCC cells

**Figure 5.**
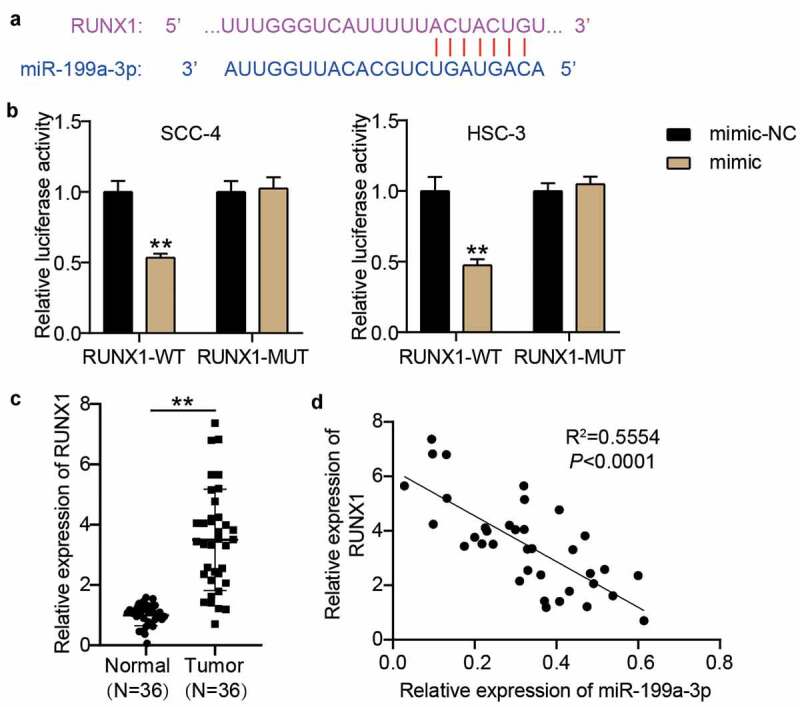
RUNX1 was a target of miR-199a-3p. (a) The binding sites between RUNX1 3ʹUTR and miR-199a-3p were predicted by TargetScan. (b) The relationship between RUNX1 and miR-199a-3p was detected by luciferase assay. WT, wild-type. MUT, mutant. mimic, miR-199a-3p mimic. NC, negative control. **P < 0.001. (c) The expression of RUNX1 in normal tissues and tumor tissues. Tumor, OSCC tissues. Normal, adjacent normal tissues. **P < 0.001. (d) The correlation between miR-199a-3p expression and TTN-AS1 expression in OSCC tissues

According to the prediction of starBase, there were binding sites between RUNX1 3ʹUTR and miR-199a-3p (Figure 5a). After constructing the RUNX1-WT and RUNX1-MUT vectors, the luciferase assay revealed that the luciferase activity in the RUNX1-WT and miR-199a-3p mimic groups decreased by approximately 50% in SCC-4 and HSC-3 cells, whereas the luciferase activity in the other groups did not significantly change (Figure 5b). The RT-qPCR assay showed that RUNX1 expression was elevated in OSCC samples (3.50 ± 1.66) compared with adjacent normal samples (1.00 ± 0.346) (Figure 5c), and its expression was negatively correlated with miR-199a-3p expression in OSCC samples (Figure 5d).

### The negative effect of si-RUNX1 was partly relieved by miR-199a-3p inhibitor in OSCC cells

**Figure 6.**
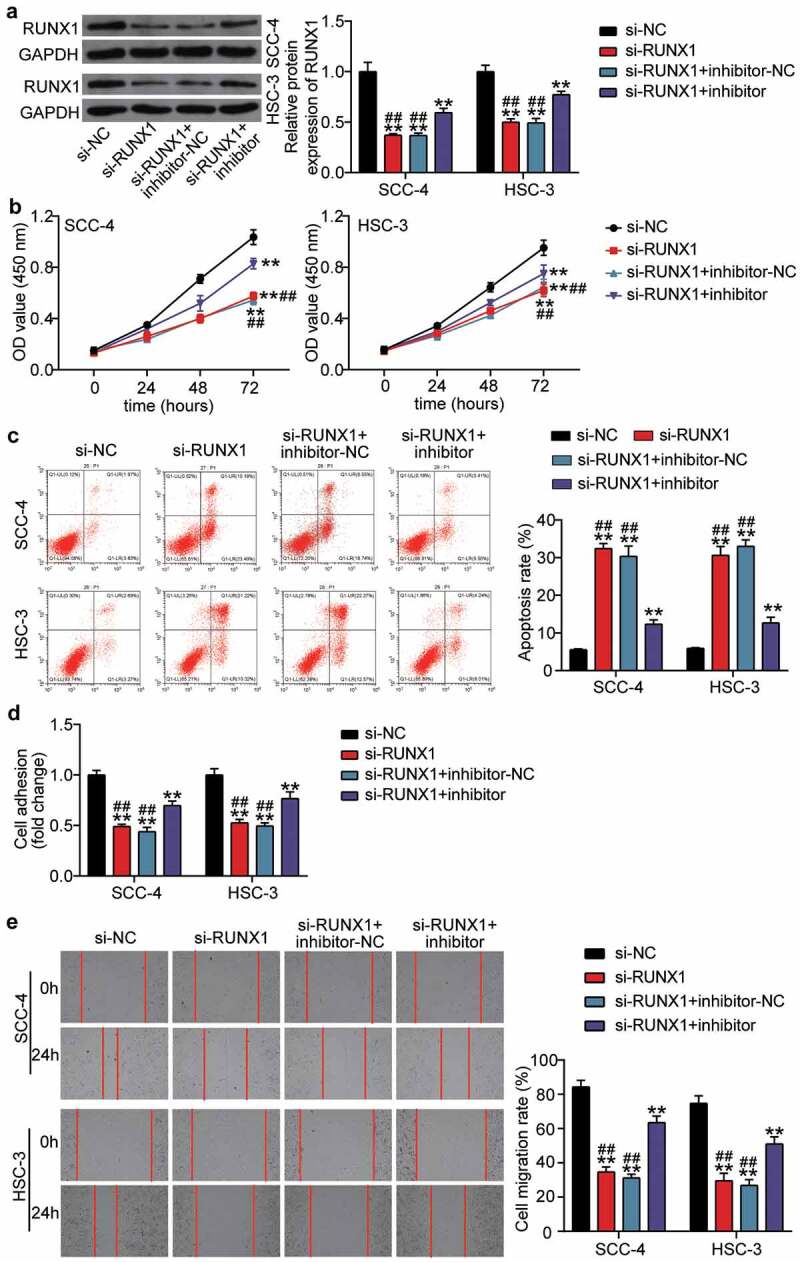
The negative effect of si-RUNX1 was partly relieved by miR-199a-3p inhibitor in OSCC cells (a) The expression of RUNX1 protein in SCC-4 and HSC-3 cells was detected by western blotting. (b) The negative effect of si-RUNX1 on cell proliferation was partly relieved by miR-199a-3p inhibitor. (c) The positive effect of si-RUNX1 on cell positive was partly relieved by miR-199a-3p inhibitor. (d) The negative effect of si-RUNX1 on cell adhesion was partly relieved by miR-199a-3p inhibitor. (e) The negative effect of si-RUNX1 on cell migration was partly relieved by miR-199a-3p inhibitor. inhibitor, miR-199a-3p inhibitor. NC, negative control. **P < 0.001 vs. si-NC. ##P < 0.001 vs. si-lnc+inhibitor

We transfected si-RUNX1 into SCC-4 and HSC-3 cells to identify the effect of RUNX1 due to the high expression of RUNX1 in OSCC. Western blotting showed that si-RUNX1 decreased RUNX1 protein expression by ~ 50%, whereas the negative role of si-RUNX1 in RUNX1 protein expression was partly inhibited by co-transfection with miR-199a-3p inhibitor (Figure 6a). After performing the CCK8 assay, it was found that silencing RUNX1 impaired cell proliferation in OSCC cells, but miR-199a-3p inhibitor relieved this negative effect (Figure 6b). The results from flow cytometry suggested that the apoptosis rate was elevated by si-RUNX1 transfection, and co-transfection with miR-199a-3p inhibitor effectively inhibited the high apoptosis rate caused by si-RUNX1 (Figure 6c). The cell adhesion assay revealed that knockdown of RUNX1 reduced cell adhesion by ~50% compared with that in the si-NC group, whereas co-transfection of si-RUNX1 and miR-199a-3p partly promoted cell adhesion compared with the si-RUNX1 group (Figure 6d). For cell migration, the wound healing assay revealed that the low migration rate caused by si-RUNX1 could be elevated by co-transfection with si-RUNX1 and miR-199a-3p (Figure 6e).

## Discussion

lncRNAs play crucial roles in cancer progression by sponging miRNAs [12,34]. This study revealed that TTN-AS1 facilitates cell proliferation, cell adhesion, and cell migration, but induces apoptosis in OSCC cells by sponging miR-199a-3p. In addition, RUNX1, a target of miR-199a-3p, had the same effect as TTN-AS1 in OSCC cells. Thus, our findings revealed the positive role of TTN-AS1 in OSCC cells by sponging the miR-199a-3p/RUNX1 axis.

The tumorigenic function of TTN-AS1 has been explored in multiple cancers, including osteosarcoma [35], lung adenocarcinoma [36] and colorectal cancer [37]. In OSCC, Fu et al. [15] demonstrated the overexpression of TTN-AS1 in OSCC tissues, and silencing TTN-AS1 restrained cell malignancy of OSCC cells by targeting the miR-411-3p/NFAT5 axis. Our study further confirmed that silencing TTN-AS1 inhibited cell proliferation and migration and induced cell apoptosis, which is consistent with the findings of Fu et al. However, we also demonstrated the positive effect of TTN-AS1 on cell adhesion in OSCC cells. In addition, TTN-AS1 has been shown to promote breast cancer progression by different miRNA/mRNA axes, including miR-524-5p/RRM2 [16] and miR-139-5p/ZEBI [17], which suggests that the regulatory mechanism of TTN-AS1 in OSCC progression might be involved in multiple molecules. Here, we used bioinformatics analysis that was not used in the study by Fu et al. [15] to predict the downstream of TTN-AS1, and the results of bioinformatics analysis and cell functional experiments showed that the miR-411-3p/NFAT5 axis was another downstream effector of TTN-AS1 in OSCC. Thus, our study enriches the regulatory mechanism of TTN-AS1 in OSCC progression.

miR-199a-3p has been shown to exert different functions in different cancers. In gastric cancer, miR-199a-3p targeting ETNK1 may contribute to the malignancy of gastric cancer cells [38]. In clear cell renal cell carcinoma, miR-199a-3p was shown to be downregulated in tumor tissues, and miR-199a-3p overexpression inhibited cell proliferation, invasion, and migration [39]. However, the function of miR-199a-3p in OSCC has not yet been investigated. Here, we found low expression of miR-199a-3p in OSCC tissues, and miR-199a-3p sponged by TTN-AS1 or targeting RUNX1 could partly attenuate the positive roles of TTN-AS1 and RUNX1 in OSCC cells.

Using bioinformatics analysis, we found that RUNX1 was related to cell adhesion and migration, and might be downstream of the TTN-AS1/miR-199a-3p axis. After reviewing the literature, it was found that RUNX1 is related to multiple cancer progression [40]. For instance, the RUNX1 with high expression in renal cell carcinoma is related to poor prognosis [41]. in ovarian, skin, endometrial, and epithelial cancer, RUNX1 was proved to be an oncogene [28,42–44]. In this study, we revealed the carcinogenic function of RUNX1 in OSCC cells by regulating cell proliferation, cell adhesion, cell migration, and cell apoptosis. In addition, we found that RUNX1 was downstream of TTN-AS1, which is consistent with the study of Chang et al. [45] in glioma cells that TTN-AS1 could regulated RUNX1 by sponging miR-27b-3p. However, we found that TTN-AS1 could sponge miR-199a-3p to enhance RUNX1 expression in OSCC, which is different from the study by Chang et al. in glioma.

Our study revealed the positive effect of TTN-AS1 on OSCC cells by regulating the miR-199a-3p/RUNX1 axis. However, this study has several limitations. First, our findings have been proven in vitro, but appropriate animal experiments, such as establishing a xenograft model [46], are also necessary to further prove our results in vivo. Next, our study indicates that TTN-AS1 expression is associated with tumor differentiation, T-stage, and lymph node metastasis, but we did not explore the correlation between TTN-AS1 expression and prognosis of OSCC due to the recently collected tissues. We will keep eyes for these patients to explore the effect on survival in the future, similar to the team of Xia et al. [47]. Moreover, Sarode et al. [48] explored the effect of adipose tissue on the submucosal adipose tissue of the oral cavity, suggesting that the influence of the adipose microenvironment on the treatment of OSCC may be an effective method to combat drug resistance. This may be explored in future studies. Moreover, recent studies on molecular biomarkers and alterations in gene networks in animal and human models [49] or exploring the correlation between the tumor microenvironment and the malignant properties of cancer stem cells [50] may also provide a novel direction for OSCC therapy.

## Conclusion

Our study uncovered the oncogenic roles of TTN-AS1 and RUNX1 in OSCC cells, and TTN-AS1 could sponge miR-199a-3p to enhance RUNX1 expression. Our results provide a novel target for OSCC therapy.

## Supplementary Material

Supplemental Material

## Data Availability

The data used to support the findings of this study are available from the corresponding author upon request.

## Highlights

- TTN-AS1 contributes to the OSCC progression by regulating miR-199a-3p/RUNX1 axis
- RUNX1 targets miR-199a-3p and promotes the OSCC progression
- miR-199a-3p partly relieves the effects of TTN-AS1 and RUNX1 on OSCC cells
