# Supplementary material for: Long non-coding RNA TTN-AS1/microRNA-199a-3p/runt-related transcription factor 1 gene axis regulates the progression of oral squamous cell carcinoma
Source: Bioengineered. 2021 Oct 4;12(1):7724–36. doi: 10.1080/21655979.2021.1982324 (PMC8806903; doi:10.1080/21655979.2021.1982324)
Supplement: Supplemental Material [file KBIE_A_1982324_SM9329.zip › supplementary/Supplementary Table 1_revised.docx]

Supplementary Table 1. Clinical characteristics of the study population (N=36).

| Characteristics | Number of patients | Percentage (%) | TTN-AS1 expression | | P-value |
| --- | --- | --- | --- | --- | --- |
|  |  |  | low (n=18) | high (n=18) |  |
| Age |  |  |  |  | 0.4998 |
| <60 | 15 | 41.67 | 9 | 6 |  |
| ≥60 | 21 | 58.33 | 9 | 12 |  |
| Sex |  |  |  |  | 0.4887 |
| Male | 23 | 63.89 | 13 | 10 |  |
| Female | 13 | 36.11 | 5 | 8 |  |
| Tumor differentiation |  |  |  |  | 0.0010 |
| Well | 17 | 47.22 | 13 | 4 |  |
| Moderate | 10 | 27.78 | 5 | 5 |  |
| Poor | 9 | 25 | 0 | 9 |  |
| T-stage |  |  |  |  | 0.0369 |
| T2 | 12 | 33.33 | 9 | 3 |  |
| T3 | 14 | 38.89 | 7 | 7 |  |
| T4 | 10 | 27.78 | 2 | 8 |  |
| Lymph-node metastasis |  |  |  |  | 0.0027 |
| Yes | 25 | 69.44 | 8 | 17 |  |
| No | 11 | 30.56 | 10 | 1 |  |
