# Supplementary material for: Long non-coding RNA TTN-AS1/microRNA-199a-3p/runt-related transcription factor 1 gene axis regulates the progression of oral squamous cell carcinoma
Source: Bioengineered. 2021 Oct 4;12(1):7724–36. doi: 10.1080/21655979.2021.1982324 (PMC8806903; doi:10.1080/21655979.2021.1982324)
Supplement: Supplemental Material [file KBIE_A_1982324_SM9329.zip › supplementary/Supplementary Table 2.docx]

| Gene name | Primer sequence |
| --- | --- |
| TTN-AS1 | Forward: 5ʹ-GCCAGGTAGAGTTGCAGGTT-3ʹ |
|  | Reverse: 5ʹ-GAAGCTGCTGCGGATGAATG-3 |
| miR-199a-3p | Forward: 5′-GCGGCGGACAGTAGTCTGCAC-3′ |
|  | Reverse:5′-ATCCAGTGCAGGGTCCGAGG-3′ |
| miR-200a-3p | Forward:5ʹ-TAACACTGTCTGGTAACGATGT-3′ |
|  | Reverse: 5ʹ-CATCTTACCGGACAGTGCTGGA-3′ |
| miR-199b-3p | Forward:5ʹ-AACACGTGACAGTAGTCTGCA-3′ |
|  | Reverse:5ʹ-GTCGTATCCAGTGCAGGGT-3′ |
| miR-27a-3p | Forward:5ʹ-CGCGTTCACAGTGGCTAAGT-3′ |
|  | Reverse:5ʹ-GTGCAGGGTCCGAGGTATTC-3′ |
| miR-27b-3p | Forward:5ʹ-ACACTCCAGCTGGG TTCACAGTGGCTAAG-3′ |
|  | Reverse:5ʹ-ATCCAGTGCAGGGTCCGAGG-3′ |
| RUNX1 | Forward:5ʹ-AGTGGAAGAGGGAAAAGC-3′ |
|  | Reverse:5ʹ-ATCCACTGTGATTTTGATGG-3′ |
| GAPDH | Forward: 5ʹ-AGGTCGGTGTGAACGGATTTG-3ʹ |
|  | Reverse: 5ʹ-TGTAGACCATGTAGTTGAGGTCA-3ʹ |
| U6 | Forward:5′-CTCGCTTCGGCAGCACA-3′ |
|  | Reverse:5′-AACGCTTCACGAATTTGGT-3′ |

Supplementary Table2. The sequences of PCR primers used in this study.
