# Supplementary figures and images for: Long non-coding RNA TTN-AS1/microRNA-199a-3p/runt-related transcription factor 1 gene axis regulates the progression of oral squamous cell carcinoma
Source: Bioengineered. 2021 Oct 4;12(1):7724–36. doi: 10.1080/21655979.2021.1982324 (PMC8806903; doi:10.1080/21655979.2021.1982324)

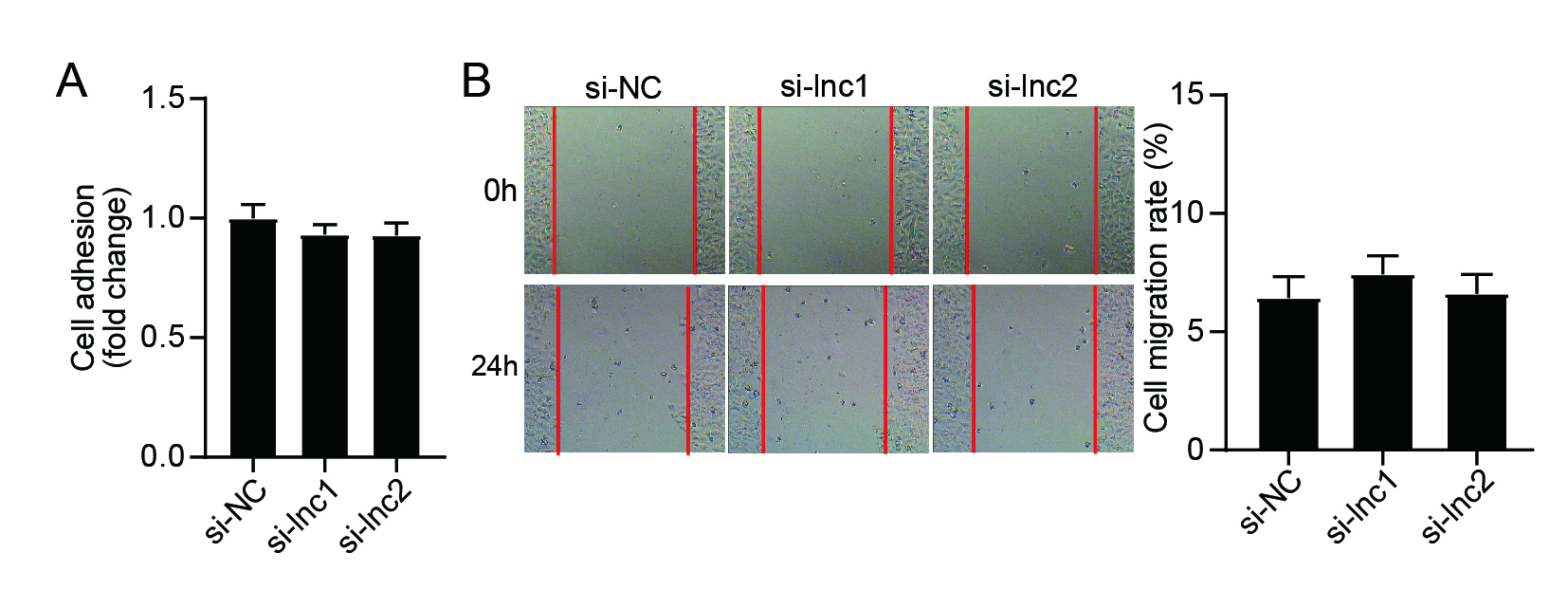

Supplement: Supplemental Material [file KBIE_A_1982324_SM9329.zip › supplementary/Supplementary Figure 1_revised.jpg]
